# Partitioning the Proteome: Phase Separation for Targeted Analysis of Membrane Proteins in Human Post-Mortem Brain

**DOI:** 10.1371/journal.pone.0039509

**Published:** 2012-06-22

**Authors:** Jane A. English, Bruno Manadas, Caitriona Scaife, David R. Cotter, Michael J. Dunn

**Affiliations:** 1 Proteome Research Centre, UCD Conway Institute of Biomolecular and Biomedical Research, University College Dublin, Dublin, Ireland; 2 Department of Psychiatry, Royal College of Surgeons in Ireland, Dublin, Ireland; 3 Proteomics Unit, Centre for Neuroscience and Cell Biology, University of Coimbra, Coimbra, Portugal; Shantou University Medical College, China

## Abstract

Neuroproteomics is a powerful platform for targeted and hypothesis driven research, providing comprehensive insights into cellular and sub-cellular disease states, Gene × Environmental effects, and cellular response to medication effects in human, animal, and cell culture models. Analysis of sub-proteomes is becoming increasingly important in clinical proteomics, enriching for otherwise undetectable proteins that are possible markers for disease. Membrane proteins are one such sub-proteome class that merit in-depth targeted analysis, particularly in psychiatric disorders. As membrane proteins are notoriously difficult to analyse using traditional proteomics methods, we evaluate a paradigm to enrich for and study membrane proteins from human post-mortem brain tissue. This is the first study to extensively characterise the integral trans-membrane spanning proteins present in human brain. Using Triton X-114 phase separation and LC-MS/MS analysis, we enriched for and identified 494 membrane proteins, with 194 trans-membrane helices present, ranging from 1 to 21 helices per protein. Isolated proteins included glutamate receptors, G proteins, voltage gated and calcium channels, synaptic proteins, and myelin proteins, all of which warrant quantitative proteomic investigation in psychiatric and neurological disorders. Overall, our sub-proteome analysis reduced sample complexity and enriched for integral membrane proteins by 2.3 fold, thus allowing for more manageable, reproducible, and targeted proteomics in case *vs.* control biomarker studies. This study provides a valuable reference for future neuroproteomic investigations of membrane proteins, and validates the use Triton X-114 detergent phase extraction on human post mortem brain.

## Introduction

Membrane proteins are at the interface between the cell and its external environment making them instrumental in synaptic and neuronal transmission via cell adhesion, cellular trafficking, and ion transport. These processes are known to be disrupted in neuropathological disorders such as Alzheimer’s disease, Parkinson’s disease, and schizophrenia. Furthermore, membrane proteins constitute one-third of the total proteins encoded by the human genome [1] making them important pharmacological and biomarker targets for drug development. Intriguingly, greater than 60% of the major pharmaceutical drug targets are known membrane proteins [2], emphasizing their crucial role in cellular dynamics and disease processes.

Despite years of extensive research, comprehensive analysis of membrane proteins is challenging to say the least [1], [3], [4]. Integral membrane proteins are defined as transmembrane proteins, with a hydrophobic domain that interacts directly with the hydrophobic core of the lipid bilayer. Thus making analysis by conventional 2-D gel-based techniques difficult due to their poor solubility, basic pH, low molecular weight, and tendency to aggregate out of solution [5]. As a consequence, membrane protein analysis is often approached by an enrichment process followed by tryptic digestion and analysis at the peptide level by LC-MS/MS [6].

**Figure 1:**
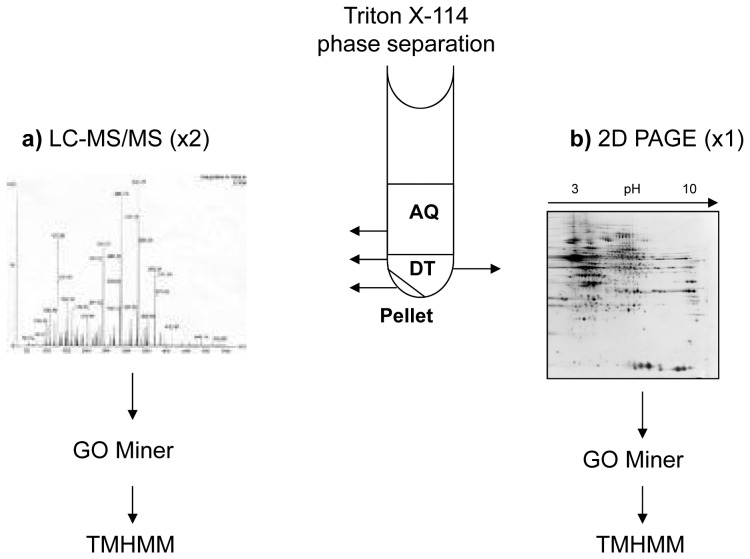
Study design for Triton X-114 phase separation on human post-mortem brain tissue. a) Following phase partitioning for two out of the three samples fractionated, Aqueous (AQ) and Detergent (DT) phase proteins, as well as proteins lost to the pellet (Pt), were identified by LC-MS/MS analysis. Identified proteins were classified according to sub-cellular location using GoMiner, and TMHMM was used to predict the number of transmembrane helices present in each protein. In b) DT and AQ phase proteins from one of the samples were isolated by acetone precipitation, resuspended in 2D-PAGE buffer, and separated by isoelectric focusing in the first dimension, and SDS- PAGE in the second dimension. The large format 2D gels underwent silver staining, and 96 of the DT phase proteins were randomly excised for identification by LC-MS/MS. Identified proteins were classified according to sub-cellular location using GoMiner, and TMHMM was used to predict the number of transmembrane helices present.

Strategies traditionally used for enriching for membrane proteins (for review see [3]), include 1) sub-cellular fractionation with a series of centrifugations, or with a sucrose density gradient centrifugation, 2) delipidation to remove the lipid bilayer surrounding the transmembrane helices, 3) affinity purification, and 4) removal of non-membrane proteins using high salt and high pH (3). These multistep protocols are often used in combination with each other to achieve sufficient power, and require large amounts of starting material. In addition, they can incur large protein losses and artifactual contamination. A fifth less documented enrichment method, phase separation, is not widely known in proteomics [7], yet it offers huge potential for routine enrichment and purification of membrane proteins prior to LC-MS/MS. Triton X-114 separation was first introduced by Bordier in the early 1980’s [8] and has traditionally been used to enrich for and study membrane proteins in bacteria [9]–[11], although more recently it has been applied to yeast [12], mouse liver [13], human cardiac tissue [6], and porcine brain [14]. To our knowledge, this is the first time phase separation using the Triton X-114 detergent has been applied to human post-mortem brain. Partitioning of the membrane and aqueous proteins is achieved by heating the Triton X-114 to temperatures above 20°C, until it reaches its cloud point. The detergent enters and partitions the lipid bilayer releasing the otherwise insoluble transmembrane proteins [6]. A simple low-speed centrifugation step recovers the membrane proteins in the detergent phase as an oily pellet, while aqueous proteins are resolved in the supernatant. As protocols with detergent/membrane combinations have not been well documented or qualified for human brain tissue, the aim of this study was to 1) perform phase separation of detergent and aqueous phase proteins in human post-mortem brain using Triton X-114, and 2) confirm enrichment for membrane proteins in the detergent phase using proteomics. The various proteomic strategies applied in this manuscript are outlined in the study design in Figure 1.

## Results

### Triton X-114 Phase Separation

Following phase separation, protein yield as determined by the Bradford dye binding assay (BioRad), was estimated at 0.45 µg/µl for the Detergent (DT) phase extract, 1.36 µg/µl for the Aqueous (AQ) phase extract, and the recovered Pellet (Pt) had 3.38 µg/µl of protein. Results are based on the average of three samples (Table S1). Each fraction was resolved in 1 ml of the appropriate buffer, giving a total of 0.45 mgs for the DT phase, 1.36 mgs for the AQ phase, and 3.38 mgs for the recovered Pt.

### 1D-SDS and Western Blotting

**Figure 2:**
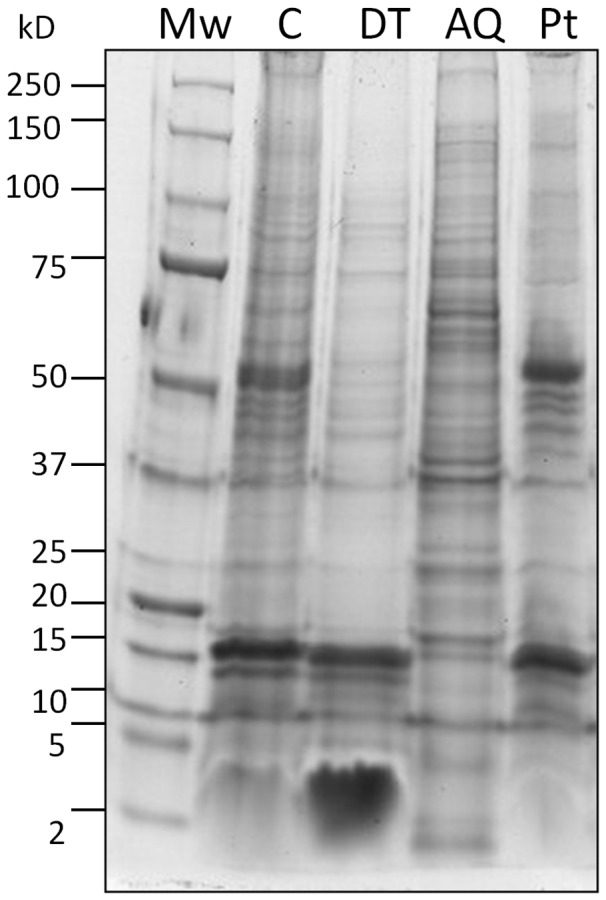
Coomassie blue staining confirming phase separation of DT and AQ phase fractions in comparison to control (C) non-enriched tissue. Proteins in the DT phase appear enriched at the low Mw region (2 kD to 15 kD) in comparison to other fractions, while protein in the 15 kD –250 kD Mw range appears depleted. The AQ fraction is much more complex with proteins distributed abundantly from high to low Mw, particularly enriched in the 15 kD to 75 kD region in comparison to the DT phase. The banding pattern for proteins recovered from the Pt is similar to that of control tissue, as expected, with strong bands at 50 kD and 15 kD.

**Figure 3:**
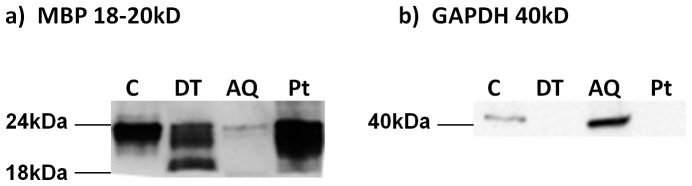
Western blotting was carried out to assess transmembrane spanning protein MBP and cytosolic protein GAPDH, on C, DT, AQ, and Pt samples. In a) an increase in MBP (18–20 kD) expression was confirmed in the DT fraction in comparison to the AQ fraction, while a considerable amount of MPB was lost to the Pt. In b) we confirmed depletion of cyotsolic protein GAPDH (40 kD) in the DT phase, as expected, and confirmed enrichment in the AQ phase. No GAPDH was lost to the Pt fraction.

Comparisons of the protein banding patterns across samples (10 µg protein/sample) revealed unique DT and AQ phase protein fractions, following phase separation (Figure 2). The DT phase was particularly enriched in protein in the low Mw region (2–15 kD) in comparison to C and AQ samples, while the medium to high Mw region (15–250 kD) was depleted with very light banding in comparison to other samples. In contrast, the AQ phase banding pattern was much more complex with proteins abundantly distributed across Mw regions. Finally, the recovered Pt exhibited patterns similar to that of C tissue with strong bands present at 50 kD and 15 kD. In support, western blotting (Figure 3) was used to show an increase in the abundant membrane associated brain protein MBP [15], [16] (18–20 kD), in the DT fraction in comparison to the AQ phase (Figure 3a). MBP was also strongly represented in the C and in the recovered Pt at 24 kD, however the banding pattern differed to that of the DT phase, where it was enriched in the 18–20 kD region (as specified in the Chemicon MAB386 data sheet), in comparison to the AQ phase. GAPDH is a well documented marker of the cytosol [17], [18], and we found this protein enriched in the AQ phase at 40 kD, as expected, and completely depleted in the DT phase and Pt fractions (Figure 3b). Together, these results suggest that phase separation of proteins into DT and AQ fractions was achieved, using the Triton X-114 technique.

### LC-MS/MS of Triton X-114 Phase Extractions

**Figure 4:**
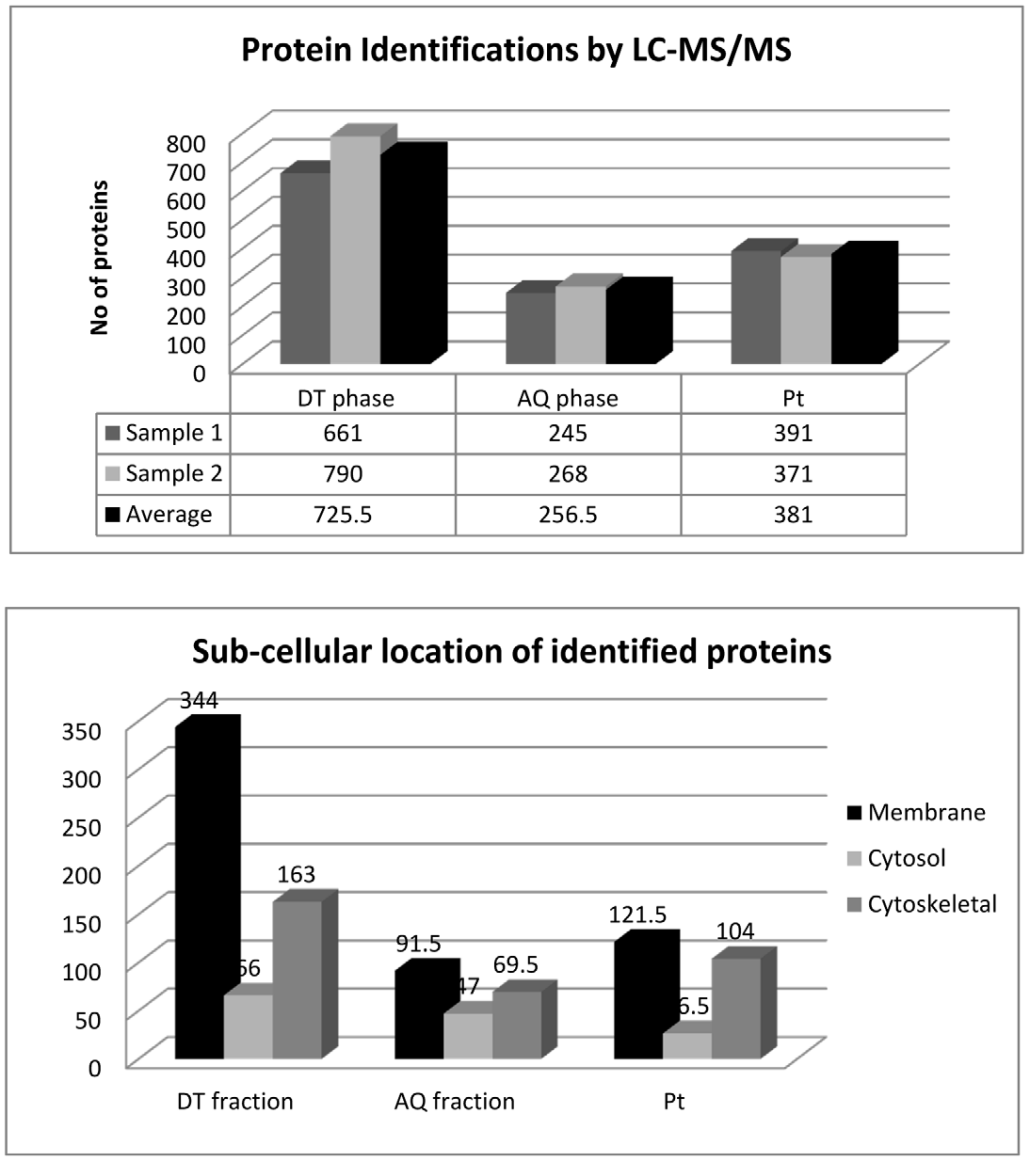
Bar chart (a) illustrates the number of proteins identified in the DT phase, AQ phase, and Pt. The reduction in the number of proteins identified in the AQ phase can be attributed to the complexity of the sample, as evident from the 1D-SDS gel in figure 3. The amount of protein present in each phase, as determined by Bradford assay, suggests that the AQ phase is more complex than that of the DT phase and Pt. Bar chart (b) summarises the sub-cellular location of the identified proteins according to membrane, cytoskeletal, and cyotsolic gene ontologies for the DT, AQ and Pt fractions. Results confirmed enrichment of membrane proteins in the DT phase, were 344 of the proteins were classified as having a membrane ontology using GO miner. Results from (a) and (b) are based on the average of two independent samples.

In order to fully characterise the protein profile of DT and AQ phase extractions, and assess the protein lost to the Pt, samples were digested to peptides and injected online to a Thermo LTQ-Orbitrap. Two independent samples from each fraction were injected on the MS. For comparative reasons, non-enriched control tissue from the same cortical brain region also underwent LC-MS/MS analysis for protein identification. Proteins were identified according to the criteria presented in the materials and methods section. On average, 726 proteins were identified in the DT phase extracts (Tables S2, S3, and S4), 257 proteins in the AQ phase (Tables S5, S6, and S7), and 382 proteins were recovered in the Pt (Tables S8 and S9). The number of identified proteins in each phase are summarised in Figure 4(a). A total of 602 proteins were identified in the control non-enriched sample from the insular cortex (Table S10). The number of identified proteins that overlapped between DT, AQ and Pt fractions for both samples are presented in Figure S1. By combining the two independent DT phase samples a total of 1154 unique proteins were identified (Table S2), including 494 (54%) membrane proteins, following the removal of 279 duplicate entries. Likewise, combined AQ phase samples revealed a total of 384 unique protein identifications, after the removal of 129 duplicate entries (Table S5).

### Gene Ontology and Transmembrane Helices Calculations

**Figure 5:**
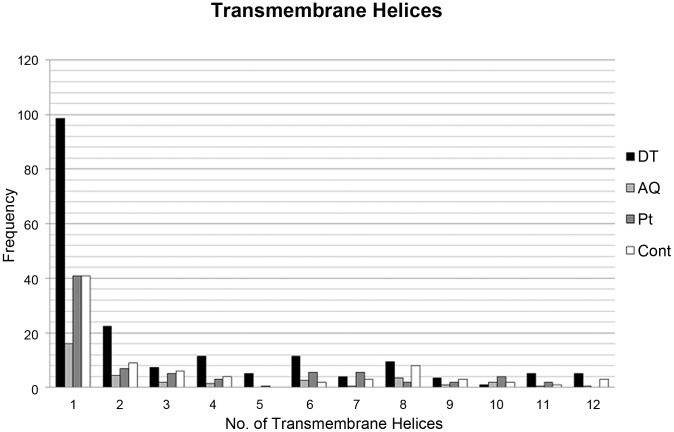
Bar chart illustrating the number of transmembrane helices present in proteins identified in the DT phase in comparison to the AQ phase, Pt, and C sample. Numbers for each fraction are based on the average of two samples. Transmembrane helices for each protein were assigned online using the TMHMM Server (v 2.0), as described in the methods section.

GoMiner was used to assign a membrane, cytosolic, or cytoskeletal sub-cellular ontology to identified proteins (Figure 4b). Gene ontology classification revealed an increase in membrane proteins present in the DT phase in comparison to the AQ phase, and the Pt. These results are based on average of two independent samples per fraction. It is also noteworthy that a large number of cytoskeletal proteins were present in the DT phase, suggesting these groups of proteins strongly associate with each other. To further characterise the properties of the DT, AQ, Pt and C samples, we used the TMHMM Server to identify the number of transmembrane spanning helices per protein, in each fraction. The number of helices present ranged from 1 to 21 for each protein. The results are summarised in Table 1, and are illustrated as a bar chart in Figure 5, which clearly shows an increased number of transmembrane domains present in the DT phase proteins in comparison to the AQ phase, protein Pt, and C sample. Most notable are the 99 proteins with at least 1 transmembrane domain, and 23 proteins with 2 transmembrane helices. Numbers of helices identified for each of the Triton-X114 phases are based on the average of two samples. DT phase proteins had an average of 194 transmembrane helices, representing a 5.5 fold increase in comparison to the average number of helices present in AQ phase proteins, and a 2.3 fold increase in comparison to control non-enriched tissue from the same brain region. Also notable is the similar number of helices present in the control sample and in protein lost to the Pt, in keeping with the similar banding patterns observed for both samples in Figure 2. This data further confirms the uniqueness of the DT phase fraction, that is abundant with large membrane spanning proteins.

**Table 1:** Summary of the number of transmembrane helices present in proteins identified in the DT phase, in comparison to the AQ phase, the recovered Pt, and control non-enriched tissue.

Predicted Helices	DT fraction	AQ fraction	Pt	Control
**1**	99	16	41	41
**2**	23	5	7	9
**3**	8	2	5	6
**4**	12	2	3	4
**5**	5	0	1	0
**6**	12	3	6	2
**7**	4	1	6	3
**8**	10	4	2	8
**9**	4	1	2	3
**10**	1	2	4	2
**11**	5	1	2	1
**12**	5	1	0	3
**13**	2	0	2	0
**14**	1	0	1	0
**15**	0	0	0	0
**16**	0	0	0	0
**17**	2	0	1	0
**18**	0	0	0	0
**19**	3	0	1	0
**20**	1	0	1	0
**21**	2	0	1	0
	194	35	83	83

Numbers for each of the Triton X-114 fractions are based on the average of two samples. Results indicate a clear increase in the number of transmembrane domains present in proteins from the DT phase, with a 2.3 fold increase (194/83) in the number of transmembrane proteins identified in comparison to control non-enriched tissue. The number of helices present in the protein lost to the Pt fraction, and in that of the control sample are very similar, as expected, and is in keeping with the protein banding pattern observed in Figure 2.

### 2D-PAGE of Triton X-114 Phase Fractions

**Figure 6:**
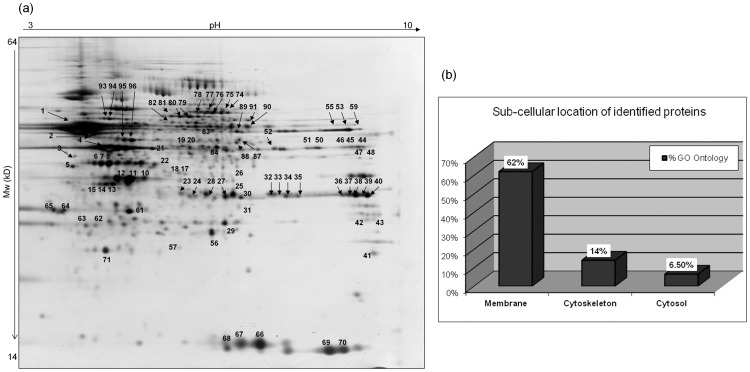
In part (a) we show a large format 2D-PAGE gel of the DT phase fraction, which was subsequently silver stained for visualisation of protein spots. We randomly excised and identified 92 of the protein spots from the 2D gel by LC-MS/MS. In part (b) the bar chart summarises the percentage of membrane, cytoskeletal, and cyotsolic proteins identified by LC-MS/MS from the DT phase gel.

2D-PAGE and subsequent silver staining of the DT and AQ phase large format gels produced good quality protein spot patterns (Figure S2), with the AQ phase being more complex and having an increased number of protein spots in comparison to the DT phase. The DT fraction had a well resolved protein spot pattern, which was surprising given the hydrophobic nature of the proteins (Figure 6). We randomly excised 96 of these membrane protein spots and successfully identified 92 by LC-MS/MS (Table S11). Of the 92 identified proteins, 77 were unique observations and could be assigned gene symbols for GO ontology classification according to sub-cellular location. Results confirmed enrichment of membrane proteins with 62% (48) of the 77 observations being of membrane protein ontology, while 14% (11) were assigned to the cytoskeleton protein class, and 6.5% (5) were assigned as having a cytosolic protein ontology. Figure 6 summarises the sub-cellular location of the membrane proteins resolved by 2D-PAGE and identified by LC-MS/MS.

## Discussion

Analysis of sub-proteomes and otherwise undetectable protein classes is becoming increasingly important in the field of neuroproteomics, where recent investigations have enriched for and studied the post-synaptic density [19], lipid rafts [20], the myelin proteome [21], [22], neuromelanin granules [23], and the calmodulin-binding proteome [24], in human post mortem brain. In this study, we propose a paradigm to enrich for and study membrane proteins in human post-mortem brain. As integral membrane proteins are at the interface between the cell and external environment, and sub-cellular structures, they are important mediators of cell-to –cell signalling, synaptic transmission, cellular transport [4], and neuroleptic activity. Analysis of this sub-proteome in patients and disease models will greatly aid pathophysiological investigations, yet such studies have not been broadly applied due to the difficulty in recovering and resolving transmembrane proteins.

We applied Triton X-114 phase separation to human cortical tissue and confirmed phase separation into DT and AQ phases by comparing the protein banding pattern between control non-enriched tissue and DT and AQ phases (Figure 2). In support, western blotting showed increased expression of transmembrane spanning protein MBP in the DT phase in comparison to the AQ phase (Figure 3), while cytosolic protein GAPDH was depleted in the DT phase and enriched in the AQ phase, as expected. Our LC-MS/MS experiment identified a total of 1154 unique DT phase proteins (Table S2), and confirmed enrichment where 54% (494) were of membrane protein ontology. DT phase proteins had an average of 194 transmembrane domains present (Figure 5), representing a 5.5 fold increase in comparison to AQ phase proteins, thus confirming phase separation. Furthermore, a 2.3 fold increase in transmembrane spanning proteins was achieved in comparisons to control non-enriched tissue from the same brain region, further confirming the method of enrichment. Proteins identified within the DT phase included glutamate receptors (GRIA2, GRIA4), vesicular glutamate transporter (VGLU1), G proteins (GPR98, GPC5B), sodium channels (SCNAA, SCN3A, SCN5A, SCN7A), voltage gated and calcium channels (VDAC1, VDAC2, VDAC3, CAC1F, CAC1B, CAC1E) synaptic proteins (SV2A, SYNJ1, SNG3, SYNP2, SNP25, STX1A, etc.), vesicle-associated membrane proteins (VAPA, VAPB, VAMP2), myelin related proteins (MBP, PLP, MOG, 2,3 CNP), septin proteins (SEPT 6–13), calcium transporting subunits, and Rab proteins (RAB 1-15), all of which are interesting targets and warrant quantitative investigation in psychiatric [25] and neurological diseases (Table S2). As this method was designed with quantification for clinical investigations in mind, it is important to note that the data obtained from this analysis is quantitative in the form of spectral counting [20] or the chromatographic peak area for relative quantification across samples [26].

In addition to membrane proteins, a large number of cytoskeletal proteins were present in the DT phase (Figure 4), which is in keeping with the large overlap in protein identifications observed between the DT and the Pt fractions (Figure S1), and with our western blot data which showed MBP to be abundantly expressed in the Pt fraction (Figure 3a). Indeed, co-localisation of membrane and cytoskeleton proteins was also observed by Donoghue PM and colleagues, who assessed Trion-X114 phase separation on human cardiac tissue [6]. On a different note, it is important to address the poor overlap in protein identifications by LC-MS/MS (i.e. 297 proteins) between the DT phase of sample 1 (661 proteins) and sample 2 (790 proteins). This is most likely due to the properties of the membrane proteins and their affinity for the column on the chromatography system, whereby increasing the number of injections would provide a more accurate representation of proteins present within each sample. In general, triplicate runs are suggested for differential expression analysis by MS [27], [28] or multiplexing the samples by labelling peptides (e.g. iTRAQ) [29] prior to MS can be carried out to overcome the notorious problem of MS reproducibility [30].

That said, we found the Trion X-114 method to be reproducible in terms of protein recovery from the three independent samples assessed, where we obtained similar protein yields for DT, AQ, and Pt fractions from the three independent samples, with the average protein yield being 0.45 µg/µl and 1.36 µg/µl for the DT and AQ phases, while 3.4 µg/µl of protein was lost to the Pt (Table S1). Protein recovery of the DT phase extract was similar to that observed in human cardiac tissue, where 0.5 µg/µl was obtained for the DT phase extract, and 5 µg/µl was extracted from the AQ phase [6]. Of the 113 proteins identified in the DT phase extract of the heart, 34% were assigned a membrane protein ontology using GO miner. Similarly, findings of a Triton-X-114 phase separation study on porcine brain identified 331 proteins in the DT phase, 27% of which were annotated as membrane proteins. In comparison, our method identified substantially more unique proteins (1154) and more membrane proteins, including 494 (54%) proteins with a membrane protein ontology using GO miner. Also, this is the first Triton-X114 study to examine the number of transmembrane helices present for the DT phase following enrichment.

LC-MS/MS analysis identified a total of 384 AQ phase proteins, where neither cytosolic, cytoskeletal, or membrane sub-cellular ontology’s were particularly prevalent (Figure 5) and the number of transmembrane helices present was dramatically reduced in comparison to DT phase proteins, with an average of 35 helices present in AQ1 and AQ2 samples. The reduced number of identifications observed in comparison to DT phase samples is likely due to 1) AQ phase sample complexity, as indicated by protein banding pattern in Figure 2, and 2) the protein yield of 1.36 mg which was considerably larger than that of the DT phase at 0.45 mg. It’s possible that a 2-Dimensional fractionation of AQ peptides, prior to MS, would improve identifications [31]. 2D-LC-MS/MS would increase the orthogonality of peptide elution, simplifying the mixture and thus increasing the number of protein identifications [32]. These results further highlight the need to pre-fractionate complex protein samples prior to in-depth LC-MS/MS analysis for successful biomarker identification.

In addition to analysis of DT and AQ phase extracts, we used LC-MS/MS to assess protein loss incurred from Triton X-114 separation, whereby the pellet from the initial ultracentrifugation step was retained and solubilised for analysis. Results indicate that 1.4% of protein is lost to the pellet, along with cell debris prior to phase separation, including several membrane and cytoskeletal proteins (Figure 4). However, it is possible to recover the proteins by introducing a wash step of the pellet and adding the “wash” back to the Triton X-114/PBS supernatant prior to phase separation.

Finally, this study examined the feasibility of profiling the DT phase proteins with traditional 2D-PAGE technology, as this is generally regarded as not suitable for resolving large transmembrane spanning proteins. Protein spots were relatively well resolved on the 2D gel, particularly in the low pH region and in the medium to high Mw regions, while the basic pH and low Mw regions were poorly populated, as expected [5]. In contrast, the AQ phase gel has protein features abundantly distributed across pH and Mw regions, with several protein spots present at the low Mw region (Figure S2). To assess DT phase proteins that entered the gel, we used LC-MS/MS to identify 92 protein spots that were randomly excised, and the sub-cellular location of the identified proteins were assessed. Of interest, 66% of the identified DT phase proteins localised to the membrane, further confirming Triton-X114 enrichment for membrane proteins. That said, only 2 out of the 92 proteins identified from the gel had transmembrane helices (Table S11), suggesting these are membrane associated proteins rather than integral membrane proteins. While the 2D-PAGE platform is therefore not recommended for the detection of transmembrane spanning proteins, the technology is very robust and reproducible, and should not be discarded lightly for analysis of more soluble sub-proteomes such as that of the AQ phase extract.

In conclusion, Triton X-114 phase separation is a simple and efficient technique for partitioning the proteome into hydrophobic and hydrophilic fractions, with minimal protein loss incurred. This pre-fractionation step offers more flexibility and control when it comes to sample complexity, as once the DT and AQ phases are recovered, the appropriate proteomic technique can be applied, based on the biochemical properties of the sub-proteome. For example, DT phase membrane proteins can be digested to peptides and samples can be multiplexed by labelling (e.g. iTRAQ) prior to LC-MS/MS analysis so technical reproducibility is no longer an issue. In contrast, AQ phase samples could be assessed by applying a 2-Dimensional technique such as 2D-PAGE or 2D-LC-MS/MS to further reduce sample complexity and improve proteome coverage. The Triton X-114 sample preparation method can be applied to a broad range of organisms and tissue types, and can have several applications. The information presented creates a valuable resource for future neuroproteomic studies, where targeted analysis of low abundant, integral, or otherwise undetectable membrane protein in desirable in the quest to find disease associated biomarkers and potential drug targets.

## Methods

### Triton X-114 Phase Separation

The phase separation was carried out in triplicate, on three independent samples, with one sample appropriately prepared for 2D-PAGE and the other two samples reserved for LC-MS/MS. Healthy human post-mortem brain tissue was obtained from the Stanley Foundation Brain Consortium (www.stanleyfoundation.com) and ethical approval (application no. 080) was granted by the Royal College of Surgeons in Ireland (RCSI) research ethics committee. Briefly, 250 mg of insular cortex was sonicated on ice, in 3×10 second bursts, in 1 ml of PBS containing protease inhibitor cocktail tablets (Roche). Once solubilised, the three samples were made up to 8 mls with ice cold PBS containing PIC’s, and 2 mls of 10% Triton X-114 was added (Calbiochem). Samples were incubated overnight at 4°C on a rotary shaker. Samples were centrifuged at 20,000×g_av_ for 30 min at 25°C to remove cell debris. The resulting pellet (Pt) was retained and solublised in 1 ml of 1 M TEAB buffer (Sigma) for MS (×2 samples) and in 2D-PAGE lysis buffer [7 M urea, 2 M thiourea, 20 mM Tris, 2% CHAPS 2% DTT, 1.6% pharmalyte, pH 8.5] in order to assess protein loss. The supernatant was placed at 37°C for 30 min to allow the Triton X-114 to reach cloud point, and the sample was spun at 5,000×g_av_ for 30 min, at 25°C, to partition the sample into detergent (DT) and aqueous (AQ) phases. The AQ top layer was removed to a fresh 50 ml tube to which 2 ml of 10% Triton X-114 was added, and the DT phase oily pellet was resuspended in 8 mls of cold PBS. Both AQ and DT phases were washed three times to remove contaminants. Acetone precipitation (80% v/v) was carried out overnight at −20°C to extract protein from both AQ and DT phases. Following a 30 minute centrifugation spin at 5,000×g_av,_ at 4°C, precipitated protein extracts were resuspended in the appropriate buffer. Fractions from one case were solubilised in standard 2D-PAGE lysis buffer and fractions from the other 2 cases were dissolved in 1 ml of 1 M TEAB for LC-MS/MS analysis. Samples were briefly sonicated to aid protein solubilisation and protein concentration was determined by the Bradford dye binding assay (BioRad), according to the manufacturer instructions.

### 1D-SDS and Western Blotting

To initially examine Triton X-114 phase partitioning, a 1D-SDS gel was run and subsequently stained with Coomassie blue to allow comparison of protein banding patterns between control non-enriched tissue (C), DT and AQ fractions, and the recovered Pt. Ten micrograms of protein from each sample was denatured and solubilized in 2× Laemmli buffer (Biorad), and separated on 4–20% gradient, 7 cm, precast acrylamide gels (Pierce). The gel was stained overnight with colloidal Coomassie Brilliant Blue G-250 (Biorad). Secondly, western blot analysis was used to assess the transmembrane spanning protein Myelin Basic Protein (MBP) and cytosolic protein GAPDH, as previously described [33]. Briefly, 5 µg of protein from C, DT, AQ, and Pt fractions were resolved on 4–20% gradient, 7 cm, precast acrylamide gels (Pierce). Blots were incubated overnight at 4°C with primary antibodies against MBP (chemicon MAB386; 1∶1000), and GAPDH (abcam; 1∶2000), as optimised. Species specific secondary antibodies were sourced from GE Healthcare (RPN2124).

### LC-MS/MS of Triton X-114 Fractions

For digestion, 50 µg of protein from DT (×2) and AQ (×2) fractions, and the Pt (×2) was dried by rotary evaporation under vacuum (SpeedVac, Savant). As a further control, 50 µg of protein from non-enriched cortical tissue was digested and run alongside the Triton X-114 fractions. Samples were resuspended in 20 µl TEAB, and denatured for 10 min at 80°C in the presence of 10 µl 2% RapiGest (Waters). Samples were reduced with 2 µl (50 mM) TCEP for 60 min at 60°C, followed by alkylation with 2 µL IAA (200 mM) for 30 min in the dark. Digestion was initiated by adding 5 µl of sequence grade modified trypsin (Promega; 1 µg/µl) to each sample, and samples were incubated overnight at 37°C on a shaker. Digestion was stopped by adding 5 µL formic acid (0.1% v/v) and samples were evaporated to dryness. Prior to LC-MS/MS analysis peptides were resuspended in 3% ACN, 0.1% FA and peptides were analysed online via the Dionex UltiMate® 3000 HPLC System and the Thermo LTQ-Orbitrap. Protein identification was performed using MASCOT with Uniprot/SwissProt release 7.6 used as the search database. Proteins with a minimum of 2 peptides and MASCOT score greater than or equal to 30 were deemed identified.

### Gene Ontology and Transmembrane Helices Calculations

The sub-cellular location of identified proteins were assigned online using GoMiner gene ontology clustering software (http://discover.nci.nih.gov/gominer/ [34]. To predict the number of transmembrane helices present in identified proteins, the swissprot accession numbers from each fraction were converted for FASTA format files with uniprot jobs (http://www.uniprot.org/jobs) and FASTA files were uploaded to the TMHMM Server v 2.0 (http://www.cbs.dtu.dk/services/TMHMM/).

### 2D-PAGE of Phase Fractions

Protein pellets from the acetone precipitated DT and AQ phase fractions were resuspended in standard 2D-PAGE lysis buffer. 2D-PAGE was carried out as we described previously (26) on pH 3–10, 24 cm immobilized pH gradient strips (GE Healthcare). For protein visualisation the PlusOne Silver Staining kit (GE Healthcare) was used with modifications to allow for subsequent identification of protein spots by mass spectrometry analysis. DT phase protein spots were excised from the 2D gel using a manual spot picker. Spot plugs were destained and proteins were digested with trypsin as we previously described [33]. Protein spots were identified by LC-MS/MS on the Agilent Q-ToF with HPLC Chip Cube interface (160 nl enrichment column, 75 µm×150 mm analytical column). Protein identification was performed using the SpectrumMill search engine (Agilent Technologies), with the IPI Human v3.61.fasta search database. Proteins with a minimum peptide score of 6, in combination a %SPI >60 were deemed identified.

## Supporting Information

Figure S1 **Venn Diagram summarising the identified proteins that overlaped between DT, AQ, and Pt fractions for both samples that underwent phase separation and LC-MS/MS analysis.**
(TIF)

Figure S2 **Proteins spot patterns from the a) DT and b) AQ phase fractions that underwent 2D-PAGE and silver staining.** The AQ phase pattern is more complex than that of the DT phase, with an increased number of protein spots at both the high and medium Mw regions, in keeping with previous observations in the protein assay, and coomassie blue staining of 1D SDS gels.
(TIF)

Table S1 **Protein assay results for DT, AQ, and Pt fractions from the three independent samples that underwent Triton X-114 phase separation.**
(XLS)

Table S2 **Unique DT proteins identified from DT1 and DT2 samples that underwent LC-MS/MS.**
(XLS)

Table S3 **Proteins identified in DT1 by LC-MS/MS.** The protein Score, Mass, and number of Peptides identified for each protein are listed in columns D, E, and F respectively.
(XLS)

Table S4 **Proteins identified in DT2 by LC-MS/MS.** The protein Score, Mass, and number of Peptides identified for each protein are listed in columns D, E, and F respectively.
(XLS)

Table S5 **Unique AQ phase proteins identified from AQ1 and AQ2 samples that underwent LC-MS/MS.**
(XLS)

Table S6 **Proteins identified in AQ1 by LC-MS/MS.** The protein Score, Mass, and number of Peptides identified for each protein are listed in columns D, E, and F respectively.
(XLS)

Table S7 **Proteins identified in AQ2 by LC-MS/MS.** The protein Score, Mass, and number of Peptides identified for each protein are listed in columns D, E, and F respectively.
(XLS)

Table S8 **Proteins identified in Pt1 by LC-MS/MS.** The protein Score, Mass, and number of Peptides identified for each protein are listed in columns D, E, and F respectively.
(XLS)

Table S9 **Proteins identified in Pt2 by LC-MS/MS.** The protein Score, Mass, and number of Peptides identified for each protein are listed in columns D, E, and F respectively.
(XLS)

Table S10 **Proteins identified in control non-enriched tissue from the Insular Cortex.** The protein Score, Mass, and number of Peptides identified for each protein are listed in columns D, E, and F respectively.
(XLS)

Table S11 **Proteins excised and identified from the silver stained 2D-PAGE gel of the DT phase extract.** A total of 77 unique proteins were identified by LC-MS/MS. Proteins marked with * have transmembrane helices present.
(XLS)

## References

[1] Santoni V, Molloy M, Rabilloud T (2000). Membrane proteins and proteomics: un amour impossible?. Electrophoresis.

[2] Hopkins AL, Groom CR (2002). The druggable genome.. Nature reviews Drug discovery.

[3] Gilmore JM, Washburn MP (2010). Advances in shotgun proteomics and the analysis of membrane proteomes.. Journal of proteomics.

[4] Rabilloud T (2009). Membrane proteins and proteomics: love is possible, but so difficult.. Electrophoresis.

[5] Gorg A, Weiss W, Dunn MJ (2004). Current two-dimensional electrophoresis technology for proteomics.. Proteomics.

[6] Donoghue PM, Hughes C, Vissers JP, Langridge JI, Dunn MJ (2008). Nonionic detergent phase extraction for the proteomic analysis of heart membrane proteins using label-free LC-MS.. Proteomics.

[7] Arnold T, Linke D (2007). Phase separation in the isolation and purification of membrane proteins.. BioTechniques 43: 427–430, 432, 434 passim.

[8] Bordier C (1981). Phase separation of integral membrane proteins in Triton X-114 solution.. The Journal of biological chemistry.

[9] Malen H, Pathak S, Softeland T, de Souza GA, Wiker HG (2010). Definition of novel cell envelope associated proteins in Triton X-114 extracts of Mycobacterium tuberculosis H37Rv.. BMC microbiology.

[10] Nally JE, Whitelegge JP, Bassilian S, Blanco DR, Lovett MA (2007). Characterization of the outer membrane proteome of Leptospira interrogans expressed during acute lethal infection.. Infection and immunity.

[11] Sinha S, Kosalai K, Arora S, Namane A, Sharma P (2005). Immunogenic membrane-associated proteins of Mycobacterium tuberculosis revealed by proteomics.. Microbiology.

[12] Everberg H, Gustavsson N, Tjerneld F (2009). Extraction of yeast mitochondrial membrane proteins by solubilization and detergent/polymer aqueous two-phase partitioning.. Methods in molecular biology.

[13] Mathias RA, Chen YS, Kapp EA, Greening DW, Mathivanan S (2011). Triton X-114 phase separation in the isolation and purification of mouse liver microsomal membrane proteins.. Methods.

[14] Shevchenko G, Sjodin MO, Malmstrom D, Wetterhall M, Bergquist J (2010). Cloud-point extraction and delipidation of porcine brain proteins in combination with bottom-up mass spectrometry approaches for proteome analysis.. Journal of proteome research.

[15] Zuchero JB, Barres BA (2011). Between the sheets: a molecular sieve makes myelin membranes.. Developmental cell.

[16] Siegel GJ AB, Albers RW (1999). Basic Neurochemistry: Molecular, Cellular and Medical Aspects..

[17] Ming M, Shea CR, Guo X, Li X, Soltani K (2010). Regulation of global genome nucleotide excision repair by SIRT1 through xeroderma pigmentosum C. Proceedings of the National Academy of Sciences of the United States of America.

[18] Grunewald TG, Kammerer U, Kapp M, Eck M, Dietl J (2007). Nuclear localization and cytosolic overexpression of LASP-1 correlates with tumor size and nodal-positivity of human breast carcinoma.. BMC cancer.

[19] Hahn CG, Banerjee A, Macdonald ML, Cho DS, Kamins J (2009). The post-synaptic density of human postmortem brain tissues: an experimental study paradigm for neuropsychiatric illnesses.. PloS one.

[20] Behan AT, Byrne C, Dunn MJ, Cagney G, Cotter DR (2009). Proteomic analysis of membrane microdomain-associated proteins in the dorsolateral prefrontal cortex in schizophrenia and bipolar disorder reveals alterations in LAMP, STXBP1 and BASP1 protein expression.. Molecular psychiatry.

[21] Ishii A, Dutta R, Wark GM, Hwang SI, Han DK (2009). Human myelin proteome and comparative analysis with mouse myelin.. Proceedings of the National Academy of Sciences of the United States of America.

[22] Taylor CM, Marta CB, Claycomb RJ, Han DK, Rasband MN (2004). Proteomic mapping provides powerful insights into functional myelin biology.. Proceedings of the National Academy of Sciences of the United States of America.

[23] Tribl F, Gerlach M, Marcus K, Asan E, Tatschner T (2005). “Subcellular proteomics” of neuromelanin granules isolated from the human brain.. Molecular & cellular proteomics : MCP.

[24] Shen X, Valencia CA, Szostak JW, Dong B, Liu R (2005). Scanning the human proteome for calmodulin-binding proteins.. Proceedings of the National Academy of Sciences of the United States of America.

[25] Gray LJ, Dean B, Kronsbein HC, Robinson PJ, Scarr E (2010). Region and diagnosis-specific changes in synaptic proteins in schizophrenia and bipolar I disorder.. Psychiatry research.

[26] Levin Y, Bahn S (2010). Quantification of proteins by label-free LC-MS/MS.. Methods in molecular biology.

[27] America AH, Cordewener JH (2008). Comparative LC-MS: a landscape of peaks and valleys.. Proteomics.

[28] Podwojski K, Eisenacher M, Kohl M, Turewicz M, Meyer HE (2010). Peek a peak: a glance at statistics for quantitative label-free proteomics.. Expert review of proteomics.

[29] Treumann A, Thiede B (2010). Isobaric protein and peptide quantification: perspectives and issues.. Expert review of proteomics.

[30] Tabb DL, Vega-Montoto L, Rudnick PA, Variyath AM, Ham AJ (2010). Repeatability and reproducibility in proteomic identifications by liquid chromatography-tandem mass spectrometry.. Journal of proteome research.

[31] Manadas B, English JA, Wynne KJ, Cotter DR, Dunn MJ (2009). Comparative analysis of OFFGel, strong cation exchange with pH gradient, and RP at high pH for first-dimensional separation of peptides from a membrane-enriched protein fraction.. Proteomics.

[32] Manadas B, Mendes VM, English J, Dunn MJ (2010). Peptide fractionation in proteomics approaches.. Expert review of proteomics.

[33] English JA, Dicker P, Focking M, Dunn MJ, Cotter DR (2009). 2-D DIGE analysis implicates cytoskeletal abnormalities in psychiatric disease.. Proteomics.

[34] Zeeberg BR, Feng W, Wang G, Wang MD, Fojo AT (2003). GoMiner: a resource for biological interpretation of genomic and proteomic data.. Genome Biol.

