# Supplementary figures and images for: Partitioning the Proteome: Phase Separation for Targeted Analysis of Membrane Proteins in Human Post-Mortem Brain
Source: PLoS One. 2012 Jun 22;7(6):e39509. doi: 10.1371/journal.pone.0039509 (PMC3382145; doi:10.1371/journal.pone.0039509)

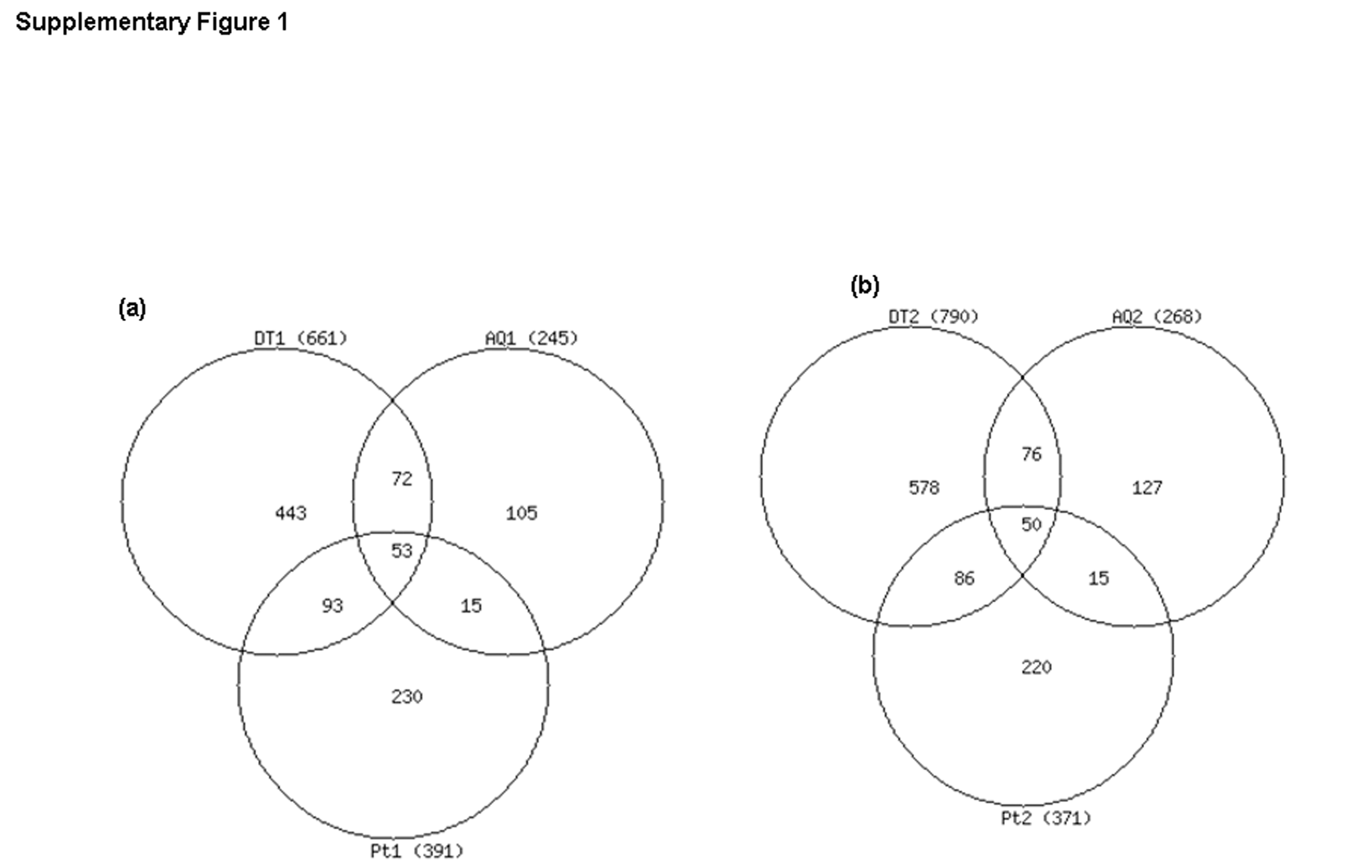

Supplement: Figure S1 — Venn Diagram summarising the identified proteins that overlaped between DT, AQ, and Pt fractions for both samples that underwent phase separation and LC-MS/MS analysis. (TIF) [file pone.0039509.s001.tif]

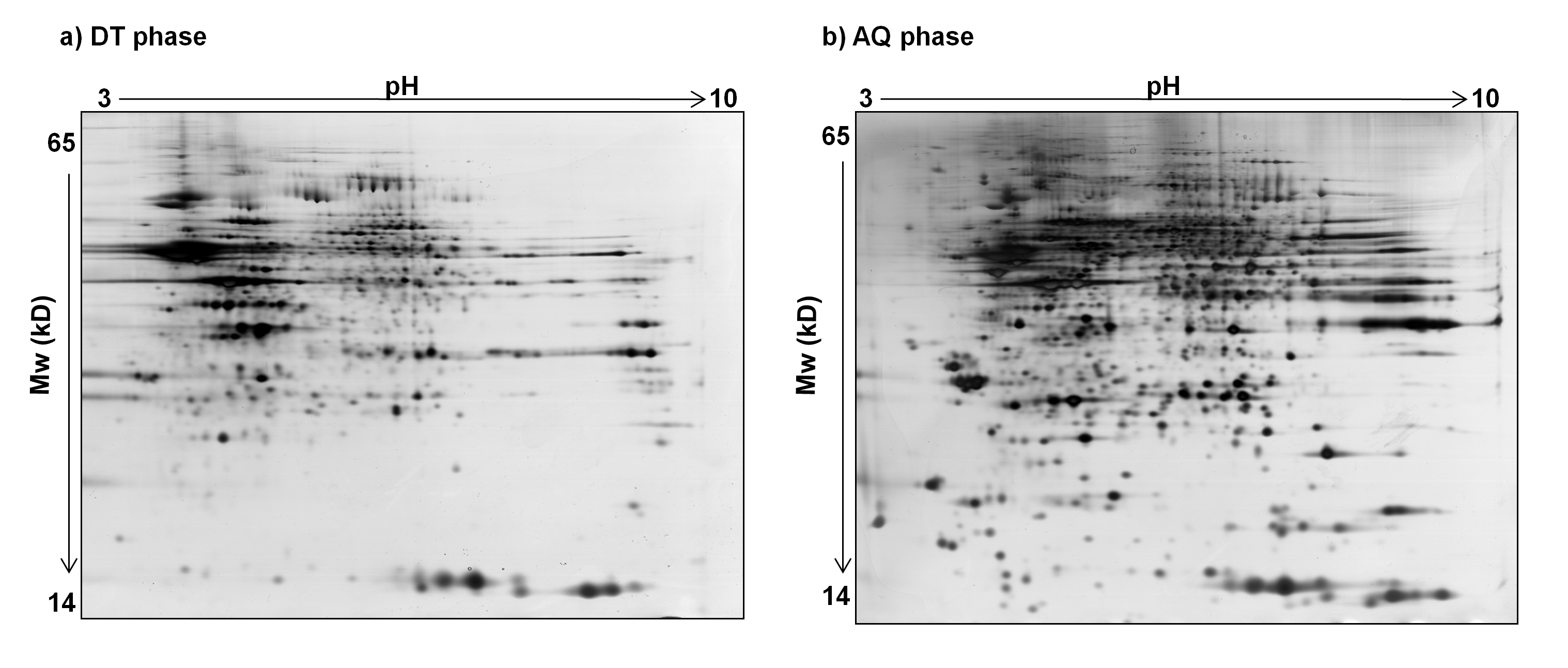

Supplement: Figure S2 — Proteins spot patterns from the a) DT and b) AQ phase fractions that underwent 2D-PAGE and silver staining. The AQ phase pattern is more complex than that of the DT phase, with an increased number of protein spots at both the high and medium Mw regions, in keeping with previous observations in the protein assay, and coomassie blue staining of 1D SDS gels. (TIF) [file pone.0039509.s002.tif]
